# Corrosion of the Welded Aluminium Alloy in 0.5 M NaCl Solution. Part 1: Specificity of Development

**DOI:** 10.3390/ma11102053

**Published:** 2018-10-21

**Authors:** Andrey S. Gnedenkov, Sergey L. Sinebryukhov, Dmitry V. Mashtalyar, Igor E. Vyaliy, Vladimir S. Egorkin, Sergey V. Gnedenkov

**Affiliations:** 1 Institute of Chemistry of FEB RAS, 159 Pr. 100-letiya Vladivostoka, Vladivostok 690022, Russia; sls@ich.dvo.ru (S.L.S.); madiva@inbox.ru (D.V.M.); igorvyal@gmail.com (I.E.V.); egorkin@ich.dvo.ru (V.S.E.); svg21@hotmail.com (S.V.G.); 2 School of Engineering, Far Eastern Federal University, 8 Sukhanova St., Vladivostok 690950, Russia

**Keywords:** aluminium alloy, weld interface, corrosion process, electrochemical measurements, cross-section

## Abstract

This work consists of two parts. In the first part, the kinetics and mechanism of corrosion on the surface of the welded joint area of the aircraft 1579 aluminium alloy have been studied using SVET (scanning vibrating electrode technique) and SIET (scanning ion-selective electrode technique) in 0.5 M NaCl. The results have revealed the corrosion process development within the weld interface due to the presence of microdefects in the morphological structure. Features of the 1579 Al alloy corrosion have also been investigated through immersion experiments, quantitative analysis of dissolved alloying elements by means of atomic absorption spectroscopy, and corrosion products characterization using XRD (X-ray diffraction) analysis. The presence of Mg as an alloying element in the 1579 Al alloy sufficiently increases the bulk pH values as a result of the intensive dissolution of Mg. These factors accelerate the corrosion activity of the studied material in the 0.5 M NaCl solution. Corrosion evolution analysis of the 1579 Al alloy sample showed the importance of the coating formation to protect this alloy against corrosion and to increase the stability of this system in the corrosive media.

## 1. Introduction

Aluminium alloys are used in various fields of industry, in automobile, construction, and engineering structures, and, especially, in the aerospace industry. The advantages of Al alloys in comparison to iron-based materials include their light weight and very good electrical and thermal conductivity. Aluminium alloys are effective materials for the reduction of construction or vehicle weight, which expands their fields of application. Pure aluminium has a high resistance to corrosion as a result of a protective film formed on its surface during exposure to air or immersion in aqueous solutions. However, pure aluminium does not have a suitable strength for most applications and, therefore, alloying elements, such as copper and magnesium, are added to improve the alloy mechanical properties. On the other hand, due to the presence of anodic and cathodic intermetallic particles in the composition of Al alloys, these materials are not sufficiently resistant to the localized corrosion process to be used for external applications without protection when exposed to aggressive media, such as marine environments [1,2,3,4,5]. In [1] Moreto et al. used electrochemical impedance spectroscopy (EIS), scanning Kelvin probe (SKP), and the scanning vibrating electrode technique (SVET) in order to assess the global and localized electrochemical behaviour of four aluminium alloys (AA2524-T3, AA2198-T851, AA7050-T7451, and AA7081-T73511) used in aircraft fabrication.

The protective properties of the welded joint of aluminium alloys inspire a great interest because of the intensive localized corrosion on its surface [6,7,8,9,10,11]. According to our earlier results [12,13,14,15,16] and works [17,18,19,20,21,22,23,24,25,26,27,28,29,30,31], the localized scanning electrochemical methods of the surface study, such as SVET and the scanning ion-selective electrode technique (SIET) are prospective methods for study of the kinetics and mechanism of the corrosion processes. Alvarez-Pampliega et al. used SVET coupled with SIET to study painted zinc metallic coated steel samples with different amounts of Al [20]. Snihirova et al. applied SVET together with localized pH measurements and oxygen distribution measurements to study the mechanisms governing the corrosion process of a sample comprising Al-Cu-Mg (S-phase in AA2024-T3) [30].

The SVET, which detects local changes of the current density due to the potential differences that occur on the surface as a consequence of the ionic fluxes generated between the anodic and cathodic sites, has become one of the most effective and widely used techniques to study the corrosion phenomena of bare and coated substrates. The SIET enables one to perform micro-potentiometric measurements with ion-selective microelectrodes, which provides information about local concentrations of specific ions (H^+^, Cl^−^, Mg^2+^, Na^+^, etc.) in solution at a quasi-constant micro-distance over an active surface in solution [32,33]. Taryba et al. used the combination of SVET and SIET to study the corrosion and self-repair processes in model defects on “smart” coatings applied to galvanized steel [32].

The 1579 Al alloy used in this work is a weldable thermally nonreinforced aluminum wrought alloy (Al-Mg-Sc system). The addition of rare-earth alloying elements to this alloy provides a mechanical properties increase compared to the base of thermally nonreinforced aluminum alloys due to the effect of structural hardening and the formation of fine-dispersed insoluble phases with aluminum. This alloy can be applied in product construction of rocket and space technology, shipbuilding, aircraft technology, and mechanical engineering [34]. The issue of lightweight Al alloys welding is topical for the application of these items in the space-rocket industry. The use of welded structures can sufficiently increase their weight efficiency (by 10–15%) in comparison to the traditional riveted type of material, which together with the use of low-density alloys, enables one to achieve a significant reduction in the weight of particular elements [35]. Preliminary studies of the 1579 Al alloy using localized methods were presented in [36] and it showed electrochemical activity in 3% NaCl solution.

This study represents an in-depth analysis of the corrosion behaviour of the 1579 Al alloy (which is commonly used in aircraft applications) with a welded joint in the chloride-containing media, and due to the large amount of experimental data collected and the great effort given to their elaboration, it is divided into two parts.

Part 1 deals with the detailed characterization of the corrosion behaviour and establishment of the corrosion destruction mechanism of the bare 1579 alloy with a welded joint area through SVET and SIET studies, immersion experiments, and corrosion products characterization. The discussion is based on quantitative parameters obtained from these localized techniques. As will be further described, the bare 1579 Al alloy system with a welded joint possesses high corrosion activity. To increase the protective properties of this system to be used in various fields of industry, Part 2 of this work is dedicated to the formation and study of the protective coatings based on the plasma electrolytic oxidation (PEO) [37,38,39,40] using SVET and SIET, as well as global traditional electrochemical techniques such as electrochemical impedance spectroscopy and potentiodynamic polarization.

The novelty of this work (Part 1) is based on the investigation of the mechanism and kinetics of the corrosion process on the surface of the welded joint zone of the 1579 aluminium alloy using SVET and SIET. Understanding the specificity of corrosion propagation will promote the development of reliable methods of welded aluminium alloy protection against corrosion and extend the area of its possible application.

## 2. Materials and Methods

### 2.1. Samples

In the present work, the 1579 aluminium alloy (6.78 wt.% Mg; 0.62 wt.% Zn; 0.14 wt.% Cu; 0.51 wt.% Si; 0.15 wt.% Fe; 0.13 wt.% Zr; 0.13 wt.% Sc; 0.17 wt.% Cr; 0.1 wt.% Ni; 0.02 wt.% Ti; balance – Al) with a welded joint was used as a sample for investigation. The welded joint was obtained using tungsten inert gas (TIG) welding of two 1579 aluminium alloy plates with a 2 mm thickness. The 1579 Al alloy parts were butt joined and welded. The wire of the 1579 aluminium alloy with a 1.0 mm diameter was used as a filler material. High-purity argon (99.999%) was used as a shield gas. The gas flow rate was 41 ± 2 L min^−1^. The interpass temperature was in the range of 55–75 °C. Welding was performed at 170 A. The TIG welding was done in accordance with the State Standard (GOST 14806-80). The weld quality estimation was carried out according to ISO 15614-2:2005 [41].

All specimens were mechanically ground for surface standardization using silicon carbide papers with the abrasive material grain size decreasing to 15 μm and, thereafter, polished with aluminium oxide paper with the grain size decreasing to 3 μm. After polishing, the specimens were washed with deionized water and dried in air.

The distribution of elements on the surface of the welded 1579 aluminium alloy was obtained using an EDX-800HS Energy Dispersive X-ray Fluorescence Spectrometer (Shimadzu, Kyoto, Japan).

### 2.2. Electrochemical Measurements

In the present work, in order to study the corrosion process on the surface of the welded joint of the 1579 aluminium alloy, the SVET/SIET system (Applicable Electronics, New Haven, CT, USA) was applied.

The probe for scanning vibrating electrode technique measurements was a Pt–Ir insulated wire with a Pt black deposited on a spherical tip of a diameter of 10 μm. The SVET probe was located at 100 μm ± 5 μm above the monitored surface and vibrated in the horizontal (X-axis) and vertical (Z-axis) planes relative to the studied surface. Frequencies of the probe vibration were 128 Hz (X-axis) and 325 Hz (Z-axis). Only the vertical (Z-axis) component was used for treating experimental data and calculating the total current [27,28,33]. The probe vibration amplitude was equal to 20 μm.

For scanning ion-selective electrode technique measurements, H^+^-selective microelectrodes were made from single-barreled glass capillaries with a 1.5 mm outer diameter. To make conic tipped capillaries using heat treatment, the P-97 Flaming/Brown Micropipette Puller (Sutter Instruments Company, Novato, CA, USA) was applied. The apex diameter of the glass capillary tip was equal to 2.0 ± 0.5 μm. After pulling, the capillaries were silanized in a glass preparation chamber at 220 °C using 200 μL of an N, N-dimethyltrimethylsilylamine injection. After silanization, the capillaries were filled with a selective ionophore-based oil-like membrane and back-filled with an inner reference solution. The membrane for the H^+^-selective microelectrode was composed of 6 wt.% 4–nonadecylpyridine, 12 mol.% potassium tetrakis (4–chlorophenyl) borate, and membrane solvent 2–nitrophenyloctyl ether [20,42]. The inner reference solution was a pH buffer made of 0.01 M KH_2_PO_4_ in 0.1 M KCl. All reagents for the pH-selective membrane were Selectophore products from Fluka (Munich, Germany).

The liquid membranes were embedded in the glass tip by means of two 3D micromanipulators and an optical microscope. The column length of the membrane of the cocktail (in the pH-microelectrodes) was about 60–70 μm. A silver chlorinated wire inserted into the internal solution was used as an inner reference electrode. The selective microelectrode was located at 50 ± 5 μm above the examined surface for SIET measurements. The H^+^-selective microelectrode was calibrated using buffer solutions in accordance with the Nernst equation. The microelectrodes demonstrated a stable and reproducible potential in the pH range 2.0–10.6 [42,43]. The Nernst slope was 57 ± 0.6 mV·pH^−1^.

An external reference electrode was a silver-chloride Ag/AgCl/0.1 M KCl, 0.01 M KH_2_PO_4_ electrode. The microelectrodes were mounted on the SVET/SIET system. The SVET/SIET measurements were controlled by means of ASET 2.0 software (ScienceWares, Falmouth, MA, USA). A preamplifier with 1 PΩ input impedance was used to measure the potential. Quasi-simultaneous SVET/SIET measurements [44] were made on the aluminium alloy samples. The distance between the SVET and SIET probes was (50 µm) – (25 µm) – (−50 µm) in X-Y-Z coordinates, respectively. The SIET probe was positioned 50 μm ahead of the SVET probe to avoid breaking the glass microelectrode due to the probe vibration and to minimize the possible cross-talk effect and solution stirring by the SVET probe.

**Figure 1:**
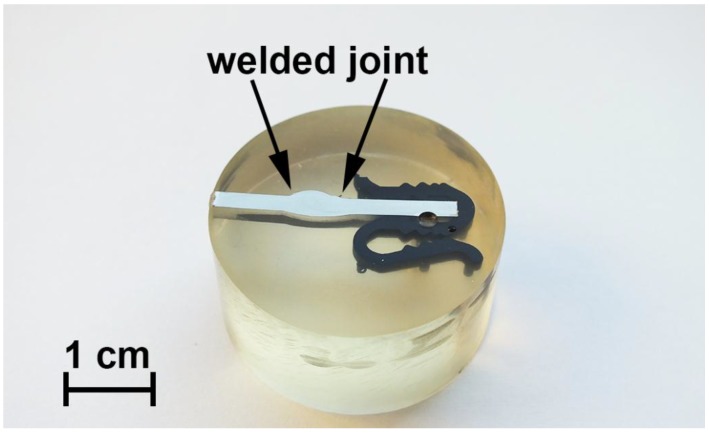
Image of the cross-section of the 1579 aluminium alloy with a welded joint zone. The sample was made for scanning vibrating electrode technique and scanning ion-selective electrode technique (SVET/SIET) measurements.

The cross-section of the welded 1579 Al alloy was made for SVET/SIET measurements (Figure 1) in order to study the effect of chloride-containing media on corrosion of the material. The investigated area of the aluminium alloy did not exceed 6 mm^2^ (after isolation with wax). In order to avoid isolation of the weld interface for the Al alloy sample, the area of the sample without wax was a little larger than that of scanning (since the weld interface is practically indiscernible before the start of the corrosion process). The local pH and ionic current density were mapped on a 31 × 31 grid. All the samples were studied in 0.5 M NaCl solution under open circuit potential conditions.

The corrosion process development on the surface of the welded joint zone of the 1579 Al alloy was monitored using SVET/SIET during specimen exposure to the corrosion-active media (up to 87 h). Since the electrolyte conductivity is of critical importance for meaningful SVET measurements, the level and concentration of the electrolyte were maintained during the entire experiment using the multiple and periodical (every 4 h) partial substitution of the old electrolyte by the new one.

The SVET/SIET tests were repeated on two similar specimens to attain reproducibility and reliability of the obtained results.

An effective method to present such a massive amount of local ionic current distribution data-maps consists of calculating total anodic and cathodic currents or the sum of these currents, and making a plot of these values versus time [20,27]. This way of data processing was proposed by McMurray and co-workers [45,46] and has been used by other researcher groups [20,23,27,47]. The evolution of total anodic and cathodic currents, as well as the sum of these currents, clearly demonstrate the activity of the sample over time and can be used to determine the stages of the corrosion development, as well as to compare different specimens.

Total anodic (*I*_anodic_) and cathodic (*I*_cathodic_) ionic current densities were calculated by integrating the current density (*i_z_*, *z* stands for the vertical axis of SVET probe vibration) distribution across the area of each scan at the time of the measurement according to Equations (1) and (2), respectively:

(1) $$\;I_{\mathrm{anodic}}=\;\int_{x_{\min}}^{x_{\max}}\int_{y_{\min}}^{y_{\max}}[i_{z}\left(x;y\right)>0]dxdy,\;$$

(2) $$\;I_{\mathrm{cathodic}}=\;\int_{x_{\min}}^{x_{\max}}\int_{y_{\min}}^{y_{\max}}[i_{z}\left(x;y\right)<0]dxdy,\;$$

where *x*_max_, *x*_min_, *y*_max_, and *y*_min_ are the coordinates of the scanned area of each sample. Thus, integrating the current density (µA·cm^−2^) and spatial dimensions of the scanned area (µm), the total cathodic and anodic currents arising from the scanned area will be expressed in µA. In the present work, we presented the plot of the sum of total anodic and cathodic currents evolution versus time.

### 2.3. Cross-Section Preparation

The cross-section of the investigated aluminium alloy with a welded joint area for SVET/SIET measurements (Figure 1) was prepared using a Tegran 25 device (Struers A/S, Denmark). The sample comprising a plate was imbedded into ViaFix acrylic resin at an angle of 90 degrees to the surface. After preliminary processing with sandpapers, the sample was ground with the MD-Largo disk using 9 μm diamond suspension and then polished with MD-Mol and MD-Nap disks using 3 and 1 μm diamond suspensions, respectively. After polishing, the sample was washed with deionized water and air-dried.

### 2.4. Immersion Experiments and Corrosion Products Characterization

A total of 0.5 M NaCl solution of pH 7.05 was used as media for exposure of the 1579 aluminium alloy with a welded joint. Immersion experiments were performed for a period of up to 30 days. The size of the sample was 25 mm × 25 mm × 1.8 mm and the volume of the electrolyte was 25 cm^3^. The electrolyte did not have contact with air. The experiment was performed at room temperature. The bulk pH of the solution was measured daily. Atomic absorption spectroscopy (AAS) (double-beam spectrometer Solaar M6 instrument (Thermo, Waltham, MA, USA)) was used to conduct a quantitative analysis of dissolved alloying elements (Mg, which will be discussed later) in the solution after 30 days of sample exposure. At the end of the immersion experiments, the sample was removed from the solution, rinsed with deionized water, and dried in air. X-ray diffraction (XRD) analysis was used to characterize the corrosion products formed on the Al alloy surface. XRD measurements of the 1579 Al samples were performed using a D8 ADVANCE diffractometer (Bruker, Billerica, MA, USA) in CuKα radiation, and the tube power was 30 kV, 30 mA at room temperature. The angle measurements were performed in the range 2*θ* = 5–90° with a step of 0.02°. All the experiments were conducted in triplicate.

## 3. Results and Discussion

### 3.1. Study of the Localized Corrosion Process on the 1579 Al Alloy Sample

For better understanding the electrochemical processes on the surface of the studied material in correlation with the composition of the alloy, element distribution mapping was obtained using the Energy Dispersive X-ray Analysis. The analysis of the results indicates the uniform distribution of the elements on the surface of the aluminium plates and welded joint area of the sample. These results indicate the possibility of a uniform propagation of the electrochemical processes on the surface of the material, which will be studied by SVET and SIET measurements.

**Figure 2:**
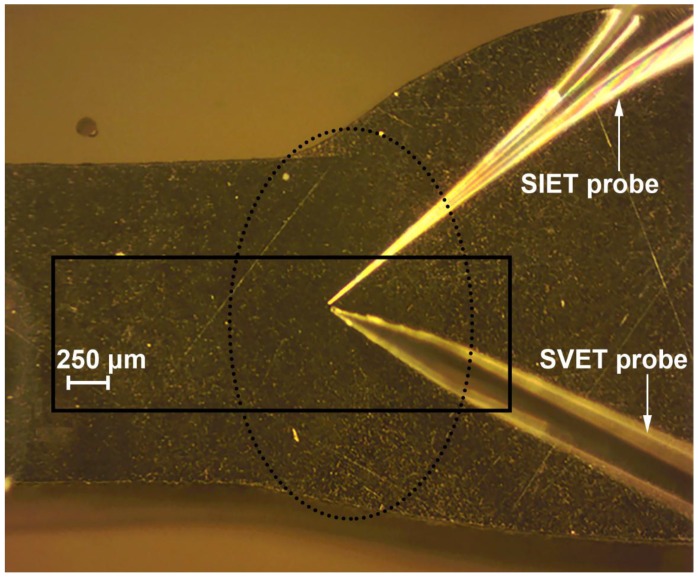
The investigated welded joint area of the 1579 aluminium alloy sample before SVET and SIET experiments. The investigated area is outlined by a frame, and the weld interface is marked by a dotted line.

**Figure 3:**
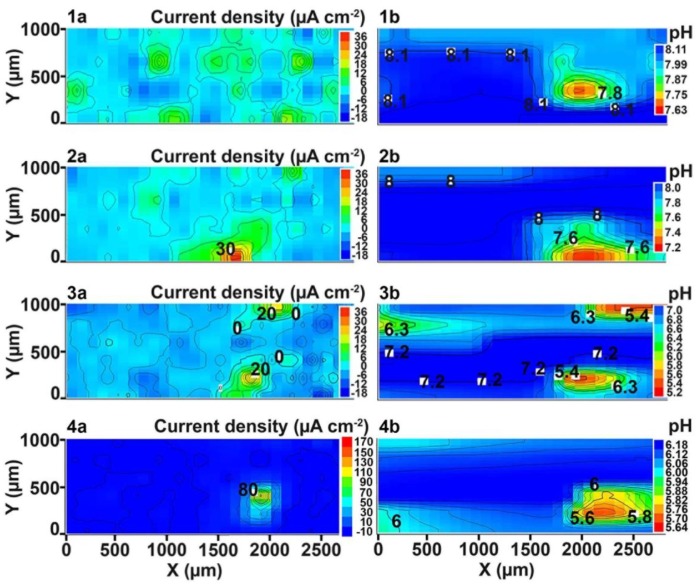
The (**a**) SVET and (**b**) SIET diagrams of the current density and pH distribution on the surface of the welded 1579 Al alloy sample after (1a,1b) 30, (2a,2b) 90, (3a,3b) 240, and (4a,4b) 510 min of exposure to 0.5 M NaCl. There is a migration of the anodic zone from the bottom part to the center (bulk of the material) of the investigated area during the exposure time.

The optical image of the investigated welded joint area by SVET and SIET is presented in Figure 2. This area is limited by frame and the weld interface of the aluminium alloy is marked by a dotted line (Figure 2). The corrosion process began to develop after 30 min of the sample exposure, according to the SVET and SIET data (Figure 3—1a,1b). There is a formation of the anodic zone (zone with lower values of the pH, red-orange area) on the weld interface (Figure 3—1b). Lower values of pH in the anodic zone for the aluminium alloy can be ascribed to the reaction of Al dissolution and hydrolysis [48]:

(3) $${\mathrm{Al}}_{\left(s\right)}+{\mathrm{nH}}_{2}O_{\left(l\right)}\to \mathrm{Al}{{\left(\mathrm{OH}\right)}_{n}^{3-n}}_{\left(s\right)}+{{\mathrm{nH}}^{+}}_{\left(\mathrm{aq}\right)}+3e^{-},\;n\;=\;1–3.$$

The aluminium ions hydrolysis reduces the local pH values of the anodic areas, making the medium more aggressive inside the pit [49], which accelerates the corrosion process. This effect leads to the accumulation of corrosion products and increases internal stresses.

**Figure 4:**
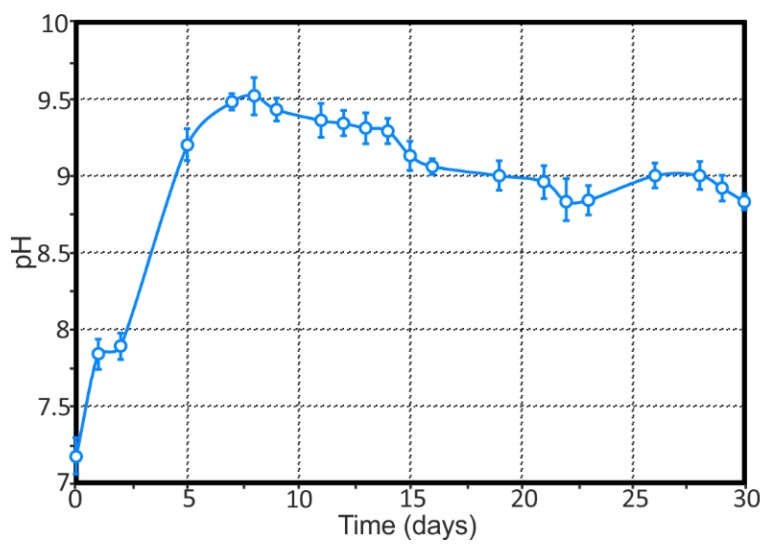
The evolution of bulk pH during 30 days of immersion experiments of the 1579 Al alloy in a 0.5 M NaCl solution. Intensive dissolution of the Mg as an alloying element of the 1579 Al alloy sufficiently shifts the bulk pH values to the alkaline range.

High values of pH in the cathodic zone (zone with lower values of the current density and with higher values of the pH, blue area) (Figure 3—1b,2b) at the initial stage of samples exposure are related to the presence of 6.78 wt.% of magnesium in the 1579 Al alloy composition. It is well-known that magnesium alloy corrosion occurs with high alkalization of the electrolyte [12,22]. The presence of the magnesium as an alloying element in a rather high amount in this sample results in alkalization of the electrolyte in the first step of the corrosion process, since Mg is a very active material. To confirm this assumption, we conducted immersion experiments of the 1579 Al alloy in 0.5 M NaCl solution for 30 days with daily monitoring of the bulk pH, according to the above-described technique. The results of this experiment are presented in the Figure 4. The first measurement of the solution pH in the presence of the Al alloy specimen was made after 15 min of the sample exposure, and this parameter was rapidly changed from 7.05 up to 7.17. There was a sharp increase of the bulk pH in solution during the first eight days from 7.05 up to 9.52. The pH decrease can be observed from nine to 22 days of sample exposure (the bulk pH decreased down to 8.83). For the remaining days, one observed a stabilization of the bulk pH values (30 days, pH = 8.83). At the end of the immersion test, AAS was used to measure the concentration of the dissolved magnesium in the solution. According to the AAS method, the concentration of the dissolved Mg was 16.5 mg·L^−1^. This result indicates intensive dissolution of Mg as an alloying element of the 1579 Al alloy, which sufficiently shifts the bulk pH values to the alkaline range (Figure 4).

**Figure 5:**
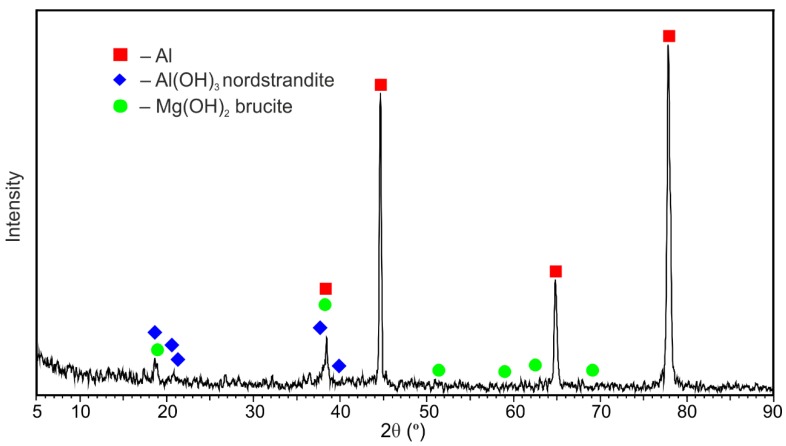
XRD pattern of the 1579 aluminium alloy sample after 30 days of immersion experiments in a 0.5 M NaCl solution. This figure shows peaks which identify Al(OH)_3_ and Mg(OH)_2_ in the composition of the corrosion products.

The X-ray diffraction measurements of the 1579 Al alloy sample at the end of the immersion tests were performed to identify the corrosion products formed on the specimen surface. Figure 5 shows the XRD pattern of the 1579 Al alloy, where one can observe peaks, which identify Al(OH)_3_ and Mg(OH)_2_ in the composition of the corrosion products.

High pH values in the cathodic area (Figure 3—1b,2b) at the initial stage of specimen exposure are also connected with the presence of dissolved oxygen in the NaCl solution, which leads, as a result of contact with the aluminium alloy substrate, to oxygen reduction as a cathodic reaction, and to dissolution of aluminium as an anodic one [49]:

O_2(g)_ + 2H_2_O_(l)_ + 4e^−^ → 4OH^−^_(aq)_ (4)

Al_(s)_ → Al^3+^_(aq)_ + 3e^−^ (5)

Therefore, higher pH values in the cathodic area are related to the reaction of oxygen reduction (4) accompanied by the secondary reaction of water reduction (6) [48]:

2H_2_O_(l)_ + 2e^−^ → 2OH^−^_(aq)_ + H_2(g)_. (6)

The global reaction of aluminium corrosion can be written as (7) or (8):

2Al_(s)_ + O_2(g)_ + 4H_2_O_(l)_ → 2Al(OH)_3(s)_ + H_2(g)_, (7)

4Al_(s)_ + 3O_2(g)_ + 6H_2_O_(l)_ → 4Al(OH)_3(s)_, (8)

with the formation of aluminium hydroxide as the main corrosion product. The possibility of the formation of Al(OH)_2_Cl and other Cl^−^-containing Al hydroxides during the anodic dissolution of pure aluminium in sodium chloride was discussed in [50]. In [20], Al(OH)_2_Cl and AlO(OH) were mentioned among the corrosion products of aluminium. It was also established [20] that Al(OH)_2_Cl and Al(OH)_3_ started precipitating at pH 2.6 and 4.8, respectively. 

The corrosion process development is presented by SVET diagrams (Figure 3—1a,2a,3a,4a). SVET began to register the anodic zone (area with higher values of the current density, red-orange zone) after 90 min of sample exposure (Figure 3—2a). The corrosion-active zone appeared in the bottom part of the investigated area (Figure 3—2a) and began to move within the weld interface during the immersion of the sample from 90 to 510 min (Figure 3—2a,3a,4a).

Analysis of the data obtained by SIET confirmed the tendency to development of the corrosion process according to SVET. There is a migration of the anodic zone to the center of the investigated area during the exposure time (Figure 3—1b,2b,3b,4b). Analysis of the results obtained by SVET/SIET indicated that the areas of the welded joint had undergone corrosion destruction. The electrochemical activity of the welded joint is a result of the presence of microdefects in the morphological structure and, as a result, microgalvanic couples formation. There are zones with different local values of the potential in the welded joint area and, therefore, the corrosion process occurs. The higher values of the current density in the SVET diagram (Figure 3—1a,2a,3a,4a) and lower values of the pH in the SIET diagram (Figure 3—1b,2b,3b,4b) confirmed the aforementioned conclusion.

An intensive corrosion process was registered at the weld interface using the SVET and SIET after 510 min of specimen exposure (Figure 3—4a,4b). The current density in this anodic area attains the value of 170 μA·cm^−2^. The pH values in the anodic area decreased from 7.6 to 5.6 (Figure 3—1b,4b), according to the reaction (4). This result indicated that the corrosion process had been passed at the weld interface of the sample at the initial stage of sample exposure (510 min).

**Figure 6:**
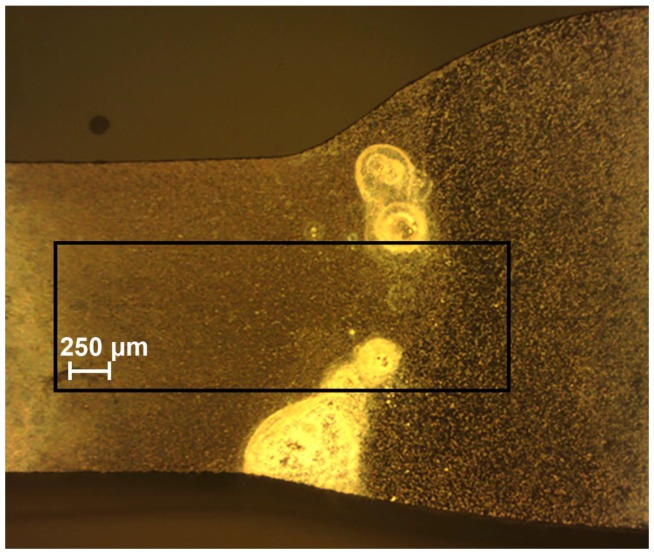
The optical image of the investigated area of the welded joint of the 1579 aluminium alloy sample after 510 min of immersion in 0.5 M NaCl solution. There is a development of the corrosion process within the weld interface.

The data obtained by SVET and SIET are in good agreement with the optical image of the investigated area after 510 min of sample exposure to 0.5 M NaCl solution (Figure 6). Analysis of the optical image (Figure 6) reveals the development of the corrosion process within the weld interface.

It should be mentioned that cross-section analysis of the sample (before immersion in 0.5 M NaCl solution) using optical microscopy did not show obvious welding defects like voids, cracks, or porosity in the weld. These microdefects were only revealed by localized electrochemical methods. The obtained results show that the most vulnerable area is the weld; more precisely, the heat affected zone [11,51]. A more detailed study on the microstructure and morphology of the weld interface is, however, outside the scope of this article.

**Figure 7:**
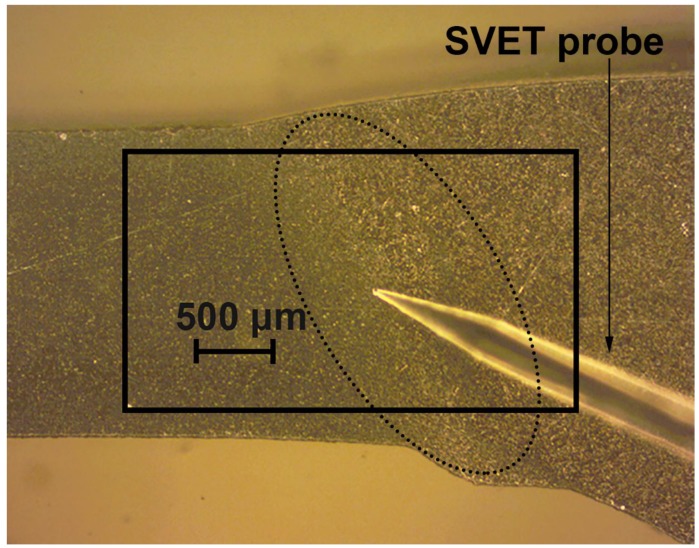
The image obtained by optical microscopy of the investigated area of the welded 1579 aluminium alloy sample before the SVET experiment for corrosion. The investigated area is outlined by a frame and the border of the weld interface of the aluminium alloy is marked by a dotted line.

In order to study the material susceptibility to corrosion, a longer-term SVET experiment was carried out. The optical image of the investigated area of the sample is presented in Figure 7. The investigated area is outlined by a frame and the border of the weld interface of the specimen is marked by the dotted line (Figure 7).

**Figure 8:**
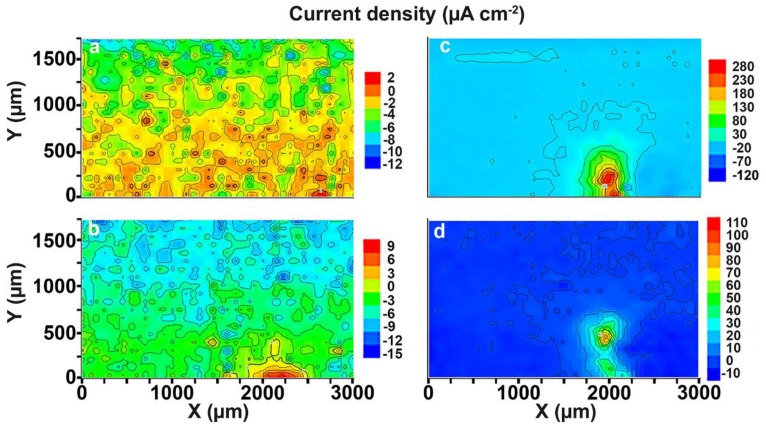
SVET diagrams of the current density values distribution on the surface of the welded joint area of the 1579 aluminium alloy sample after (**a**) 1.5, (**b**) 4.5, (**c**) 81, and (**d**) 87 h of immersion in 0.5 M NaCl. There is an anodic zone at the right bottom part of the investigated area.

Low corrosion activity was registered by the SVET over the whole surface under study after 1.5 h of sample exposure to 0.5 M NaCl solution (Figure 8a). The initiation of the corrosion process was after 4.5 h of exposure (Figure 8b). There was a formation of the anodic zone in the right bottom part of the investigated area (Figure 8b). The value of the anodic current density at this time was equal to 9 μA·cm^−2^. The intensification of the corrosion process occurred after 4.5 h of exposure. The current density in the anodic zone attained the maximum value of 280 μA·cm^−2^ after 81 h of sample exposure to chloride-containing media (Figure 8c). The intensity of the corrosion destruction in the anodic zone began to decrease after 81 h. The anodic current density values decreased to 110 μA·cm^−2^ after 87 h of sample exposure (Figure 8d). Formation and deposition of the corrosion products were observed during the entire experiment after the start of the corrosion process at the weld interface for the Al alloy sample. At the same time, after attaining the time of high intensity of the corrosion process (81 h, Figure 8c), formation and deposition of the corrosion products began to have a substantial effect on the corrosion development. Formation and accumulation of the corrosion products in the defect zone decreased the current density value from 280 to 110 µA·cm^−2^ (Figure 8c,d).

**Figure 9:**
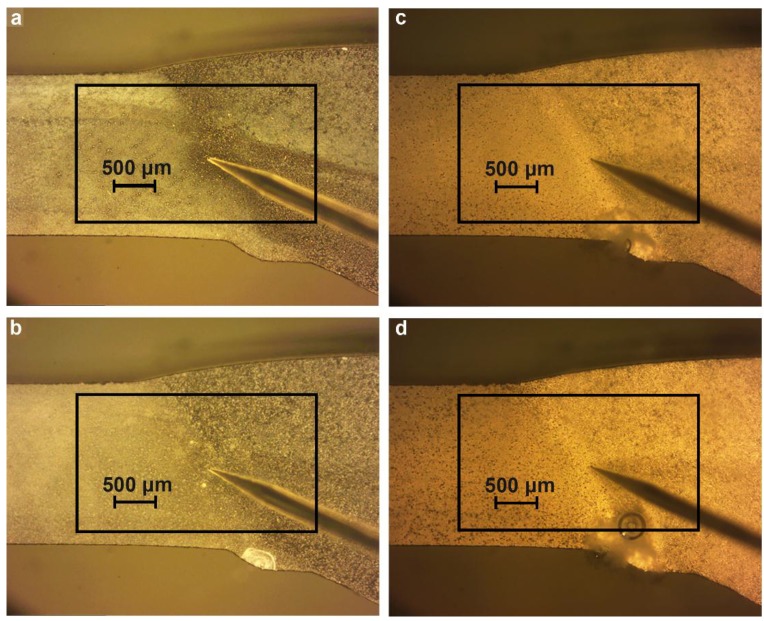
Images obtained by optical microscopy of the welded joint area after specimen exposure for (**a**) 1.5, (**b**) 4.5, (**c**) 81, and (**d**) 87 h to 0.5 M NaCl. The corrosion process started to move from the bottom part of the studied area to the bulk of the material within the weld interface.

Therefore, the reduction of the measured current density by the SVET does not necessarily mean that the corrosion activity has decreased. It can equally be that the ionic currents do not reach the SVET tip anymore, because the flux is blocked by corrosion products. Figure 9 confirms this suggestion.

It should be emphasized that the anodic zone changed its position from the bottom of the investigated area (edge of the welded joint area) to the bulk of the material during the exposure of the specimen from 81 to 87 h (Figure 8c,d).

Analysis of the images obtained by optical microscopy (Figure 9a–d) confirmed the conclusion made using the SVET. The corrosion process started in the bottom zone of the welded joint of the sample (Figure 8b) as a result of the presence of microdefects in this area (Figure 9b). After exposure of the sample for 81 h (Figure 8c, Figure 9c), the corrosion process started to move to the bulk of the material along the weld interface (Figure 8d, Figure 9d).

**Figure 10:**
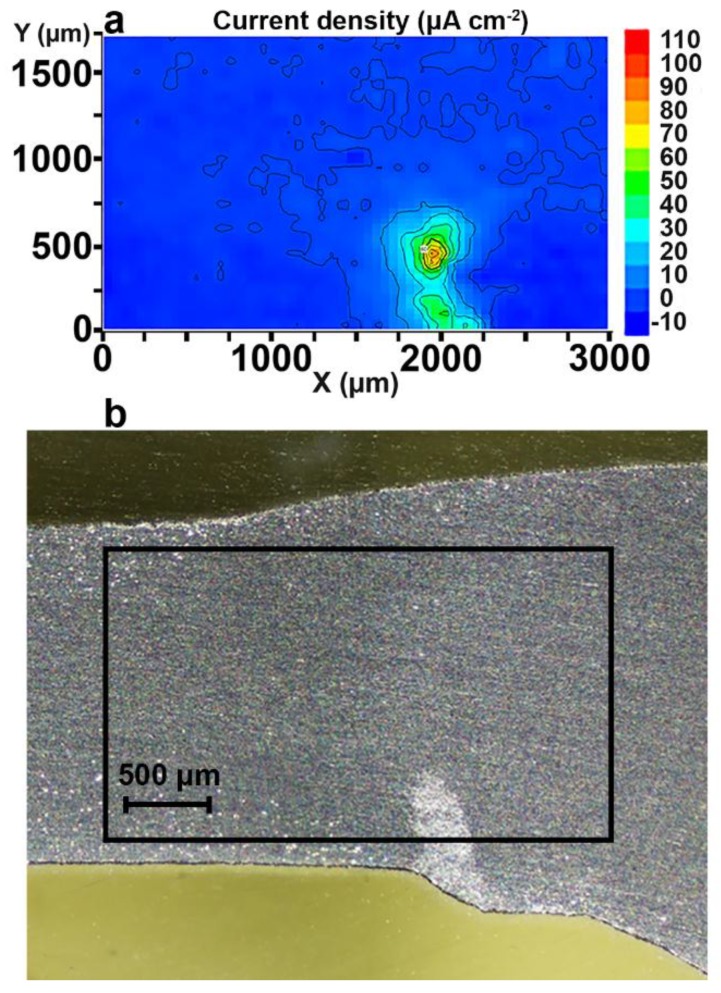
The (**a**) SVET data and (**b**) optical image of the mechanically treated sample after 87 h of immersion. The (**b**) presence of the defect in the bottom part of the welded joint area and (**a**) higher values of the current density in this zone revealed that the sample had undergone an intensive corrosion process.

The sample was mechanically ground, rinsed with deionized water, and dried with air at the end of the SVET experiment in order to remove the corrosion products formed on the surface. The defect zones that formed as a result of the corrosion destruction of the material in the chloride-containing media were studied. Analysis of the results showed the compatibility of the SVET data (Figure 10a) and optical image of the mechanically treated sample after 87 h of exposure (Figure 10b). The presence of the defect at the bottom part of the weld interface (Figure 10b) and the higher current density in this zone (Figure 10a) revealed that the sample had undergone an intensive corrosion process. One can conclude that the area of the welded joint in contact with the aggressive environment is an activator of the corrosion process, which intensifies the corrosion destruction of the studied material. Therefore, reliable corrosion protection of the aluminium alloy is required.

### 3.2. Corrosion Performance of the 1579 Al Alloy Specimen

**Figure 11:**
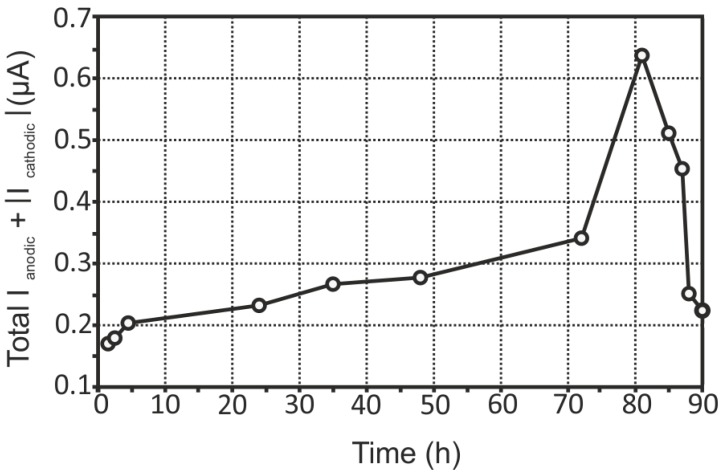
The evolution of the sum of total cathodic and anodic currents for the 1579 aluminium alloy sample with a welded joint. Increasing of the electrochemical activity was detected during the exposure of the specimen.

The evolution of the sum of total cathodic and anodic currents for the aluminium alloy sample with a welded joint is shown in Figure 11.

A definite tendency is observed for the 1579 Al alloy system: increasing of the corrosion activity was detected during the exposure of the sample. The highest values of total anodic and cathodic currents were calculated for this specimen. This sample was highly active immediately after immersion (0.17 µA after 1.5 h). A sharp increase of the corrosion activity was detected after 72 h (0.34–0.64 µA), and there was a decrease of the activity after 81 h to a value similar to the beginning of the experiment (0.22 µA) (90 h), as a result of corrosion products accumulation. These results were corroborated with SVET maps (Figure 8). 

**Figure 12:**
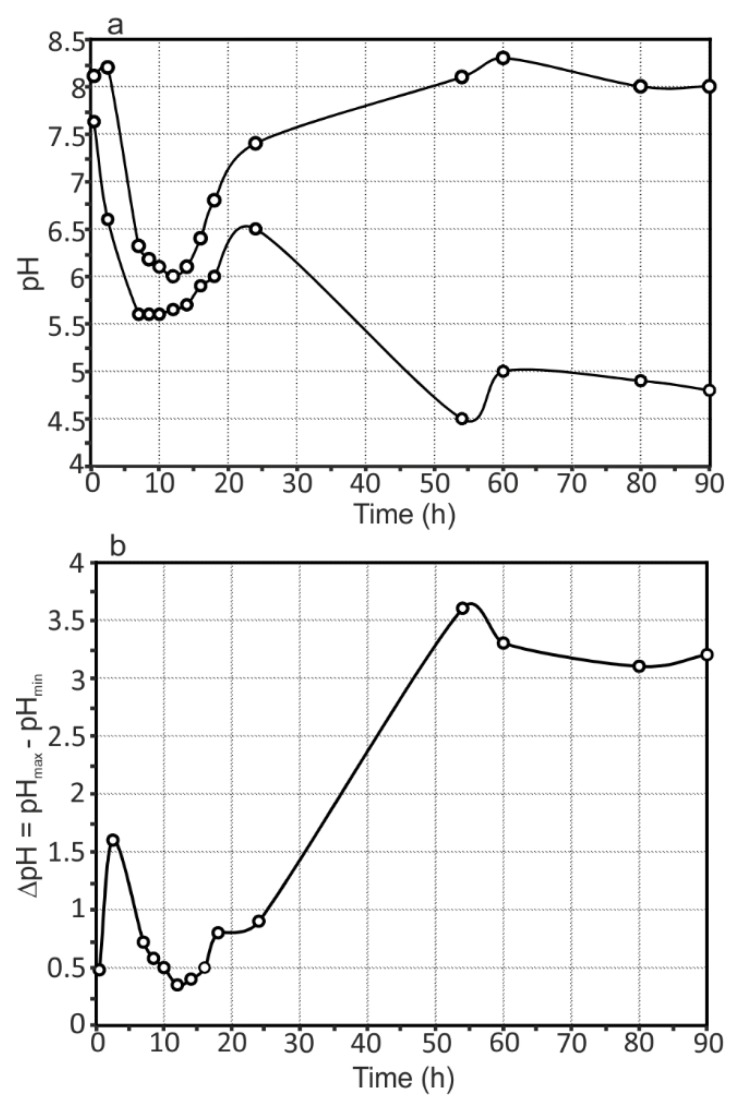
(**a**) pH _max_ and (**b**) pH _min_ as a function of time and ∆pH evolution with time for the 1579 Al alloy sample. This specimen varies very intensively, from 0.35 to 3.60, confirming the high corrosion activity of the welded 1579 aluminium alloy.

The evolution of pH distribution for the 1579 Al alloy sample (see Figure 12) can be studied by taking into account the maximum and minimum pH (pH_max_ and pH_min_, respectively) and the maximum pH difference (∆pH) between its values in anodic and cathodic areas as a function of time. The ∆pH gives an indication of the stability of the measured pH for the specimen under study. The pH dynamically changed during the experiment, thus indicating an intensive reaction of Al dissolution and hydrolysis (4), which shifts the pH to more acidic values reaching 4.5 (54 h) (Figure 12a), and intensive reactions of magnesium corrosion, which increased the pH values up to 8.3 (60 h). These results are corroborated with bulk pH measurements of the solution (Figure 4). Some discrepancy in these values (Figure 4, Figure 5, Figure 6, Figure 7, Figure 8, Figure 9, Figure 10, Figure 11 and Figure 12a) related to different methods of pH measurement, with different distances of the pH electrode from the specimen surface, different electrolyte-volume/sample-surface ratios, and multiple and periodical (every 4 h) partial substitution of the old electrolyte by the new one for SVET/SIET measurements. Analyzing the pH difference between pH_max_ and pH_min_ at each time (presented in Figure 12b), the 1579 Al alloy sample varies very intensively, from 0.35 to 3.60, confirming the high corrosion activity of the welded 1579 aluminium alloy and necessity of the coating protection. 

## 4. Conclusions

Analysis of the corrosion behaviour of the bare 1579 Al alloy with a welded joint area revealed the following conclusions:

1. Propagation of the corrosion process within the weld interface has been registered and studied by SVET and SIET. The welded joint is a zone of corrosion process activation due to the presence of microdefects in the morphological structure.
2. The presence of Mg as an alloying element in rather a high amount (6.78 wt.%) in the 1579 Al alloy sufficiently shifts the bulk pH values to the alkaline range as a result of the intensive dissolution of Mg, according to the results obtained by means of immersion tests and AAS. This effect accelerates the corrosion activity of the 1579 Al alloy in the aggressive media.
3. Al(OH)_3_ and Mg(OH)_2_ are the main corrosion products of the 1579 Al alloy, according to XRD.
4. Analysis of the evolution of the sum of total cathodic and anodic currents, as well as the evolution of pH distribution for the 1579 Al alloy sample, indicated the necessity of the coating formation to protect this Al alloy against corrosion and to increase the stability of this system in a corrosive environment.
